# Antibiotic killing of drug-induced bacteriostatic cells

**DOI:** 10.1128/aac.00156-25

**Published:** 2025-03-26

**Authors:** Teresa Gil-Gil, Brandon A. Berryhill

**Affiliations:** 1 Department of Biology, Emory University 123382, Atlanta, Georgia, USA; 2 Program in Microbiology and Molecular Genetics, Graduate Division of Biological and Biomedical Sciences, Laney Graduate School, Emory University 310203 , Atlanta, Georgia, USA; Columbia University Irving Medical Center, New York, New York, USA

**Keywords:** antibiotics, bacteriostasis, population biology, antibiotic resistance

## Abstract

There is a long-standing belief that bacteriostatic drugs are inherently antagonistic to the action of bactericidal antibiotics. This belief is primarily because the action of most bactericidal antibiotics requires the target bacteria to be growing. Since bacteriostatic drugs stop the growth of treated bacteria, these drugs would necessarily work against one another. Our results question this long-standing belief by demonstrating conditions where sequential treatment with a bacteriostatic then bactericidal antibiotic is as or more effective than treatment with a bactericidal drug alone. These results raise the need to investigate the pharmacodynamics of the joint action of bacteriostatic and bactericidal antibiotics *in vitro* and *in vivo*.

## INTRODUCTION

The clinical outcomes of antibiotic treatments involving the administration of bacteriostatic (which inhibit bacterial growth) or bactericidal (which kill bacteria) antibiotics are complex and context-dependent (1, 2). While antibiotic co-administration strategies can be beneficial in specific contexts, they require careful consideration regarding the interaction of the drugs, individualized patient factors, infection types, and resistance patterns (3). Therefore, it is necessary to evaluate specific drug combinations rather than relying solely on their classification as bacteriostatic or bactericidal. Historically, the co-administration of bacteriostatic and bactericidal antibiotics has been discouraged due to the notion that bactericidal drugs require actively dividing, metabolically active bacteria to exert their effects—an action that is directly opposed by bacteriostatic drugs. Thus, bacteriostatic drugs inhibit bacterial growth, converting “normal” cells into “bacteriostatic” cells and preventing the bactericidal antibiotic from exerting lethal effects on them (4, 5). Despite this anticipated antagonism, many multi-drug treatment regimens employed for difficult-to-treat infections use combinations of both of these drugs, often to some degree of success (6). International guidelines often recommend combining bacteriostatic drugs, such as third-generation tetracyclines (e.g., tigecycline) or oxazolidinones (e.g., linezolid), with bactericidal agents as a last-resort treatment option (7, 8). Thus, these antibiotics cannot be purely antagonistic as commonly believed. Recent work by Gil-Gil et al. has shown that, even under high concentrations of bacteriostatic drugs, cell division occurs, albeit at a rate nearly 100 times slower than in untreated cultures (9). Taken together, these previously reported results provide potential evidence and a mechanism that runs counter to the maxim that bacteriostatic and bactericidal antibiotics should not be used together.

Here, we use a laboratory strain of *Escherichia coli* (10) and a clinical isolate of *Staphylococcus aureus* (11) with combinations of bacteriostatic and bactericidal drugs to determine how pre-exposure to bacteriostatic drugs changes the dynamics of subsequent exposure with bactericidal agents. Surprisingly, we did not observe any conditions that resulted in complete antagonism between bacteriostatic and bactericidal drugs. Instead, our findings revealed a spectrum of outcomes, including delayed culture clearance, accelerated culture clearance, and even prevention of resistance emergence. These results question the maxim that bacteriostatic and bactericidal antibiotics should not be used together and raise the need to further investigate the pharmacodynamics of the use of both of these drug types together.

## RESULTS

### MIC determination and antibiotic concentration selection

**FIG 1:**
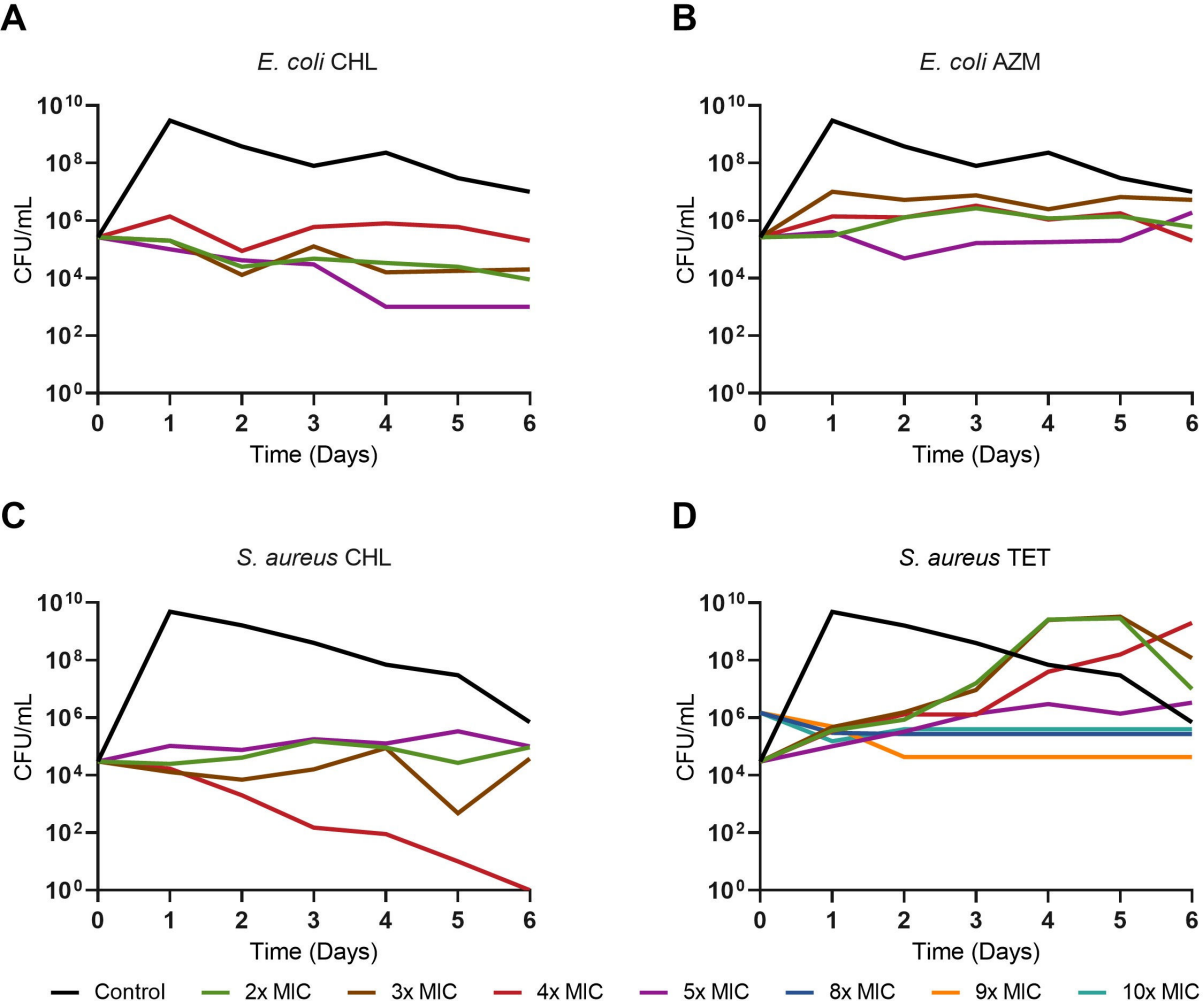
Exposure to varying concentrations of bacteriostatic drugs for 6 days without transferring. Density in CFU/mL of *E. coli* MG1655 (A and B) and *S. aureus* MN8 (C and D) measured every day for 8 days at several concentrations of either chloramphenicol (CHL) (A and C), azithromycin (AZM) (B), or tetracycline (TET) (D). Shown in black for each panel is a control culture without antibiotics.

To open our exploration of the dynamics of joint treatment with bacteriostatic and bactericidal antibiotics, we first determined the minimum inhibitory concentration (MIC) of each drug to the two bacteria used for this study, *E. coli* MG1655 and *S. aureus* MN8. The MIC values for *E. coli* were 6.25 µg/mL for chloramphenicol, 25 µg/mL for azithromycin, 0.03 µg/mL for ciprofloxacin, 12 µg/mL for gentamicin, and 25 µg/mL for ampicillin. For *S. aureus,* the MIC values were 8 µg/mL for chloramphenicol, 0.15 µg/mL for tetracycline, 0.15 µg/mL for ciprofloxacin, 1 µg/mL for gentamicin, and 256 µg/mL for ampicillin. We then determined the concentration of the bacteriostatic antibiotic that prevents net growth over the course of the 6-day experiment while not causing a substantial amount of death in these cultures (Fig. 1). Based on these results, all further experiments were conducted using 4× MIC concentrations of chloramphenicol and azithromycin for *E. coli* and 4× MIC for chloramphenicol and 10× MIC for tetracycline when using *S. aureus*. The concentration of the bactericidal drugs was selected as the lowest multiple of the MIC that does not completely eliminate the culture within 1 day.

### Exposure with a bacteriostatic then a bactericidal antibiotic

**FIG 2:**
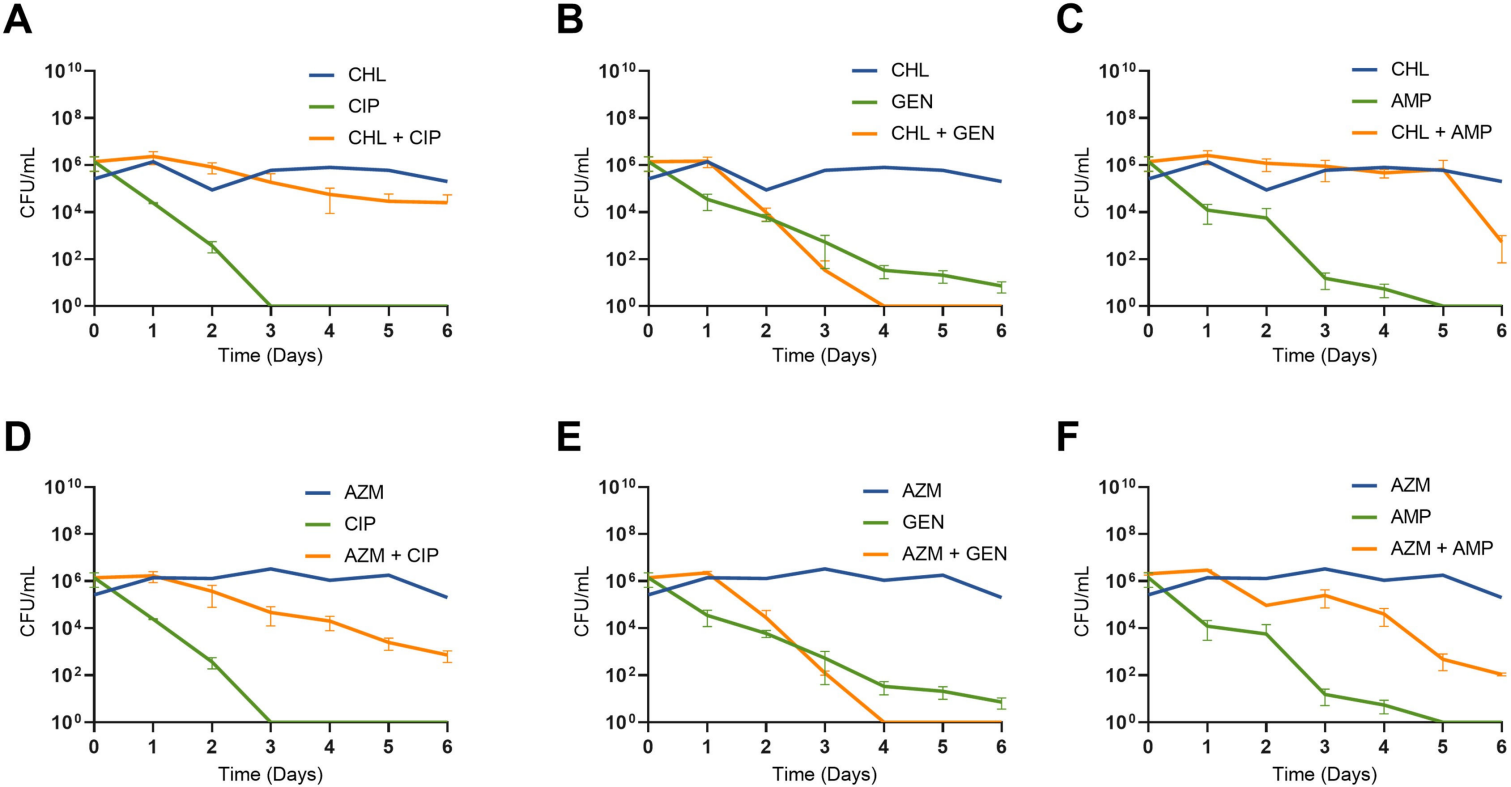
The treatment of *E. coli* with bacteriostatic followed by bactericidal antibiotics. Shown are bacterial densities in CFU/mL, each point represents the mean and standard deviation of three biological replicates. Cultures were treated with either chloramphenicol or azithromycin at day 0 before the subsequent addition of a bactericidal antibiotic at day 1 (A–F). Cultures were sampled every day for 6 days and are not transferred. Chloramphenicol is at 4× MIC, azithromycin at 4× MIC, and the bactericidal drugs are all at 4× MIC. The value of the area under the curve of cultures treated with chloramphenicol or gentamicin alone versus chloramphenicol and then gentamicin as well as azithromycin or gentamicin alone versus azithromycin and then gentamicin is significantly different by an unpaired two-tail *t*-test (****P < 0.0001). No other combinations show statistical significance.

Over the course of the experiment, the control cultures containing no drugs reached their maximum stationary phase density at 24 hours (10^9^–10^10^ CFU/mL) and then went down by one or two logs over the course of 6 days. The densities in the cultures containing the bacteriostatic drugs remained stable, as growth is occurring at the same rate as death (9). We initiated our joint action experiments by treating *E. coli* MG1655 with either chloramphenicol or azithromycin at a super-MIC concentration for 1 day. Following pre-exposure, we introduced in the same culture flask one of several bactericidal drugs with differing mechanisms of action (Fig. 2). Cultures were sampled each day over a 6-day period. At the selected drug concentrations, in every scenario where both drugs were used sequentially, the bacterial density at day 6 was consistently lower than in cultures containing just the bacteriostatic drug, indicating that bactericidal activity occurred. Notably, when gentamicin was administered after the bacteriostatic pre-exposure, bacterial clearance occurred faster than when gentamicin was used alone. This accelerated clearance is likely because, without the bacteriostatic pre-exposure, small colony variants resistant to gentamicin emerge. In the absence of pre-exposure, small colony variants began to emerge by day 3 of the experiment, representing 20% of the population, and by day 4, they had become the dominant population, constituting 100%. The pre-exposure effectively prevented the appearance of these resistant variants, thereby enhancing the overall efficacy of gentamicin. However, the dynamics of exposure with ciprofloxacin and ampicillin following the bacteriostatic drug were slower than those when either ciprofloxacin or ampicillin were used alone.

**FIG 3:**
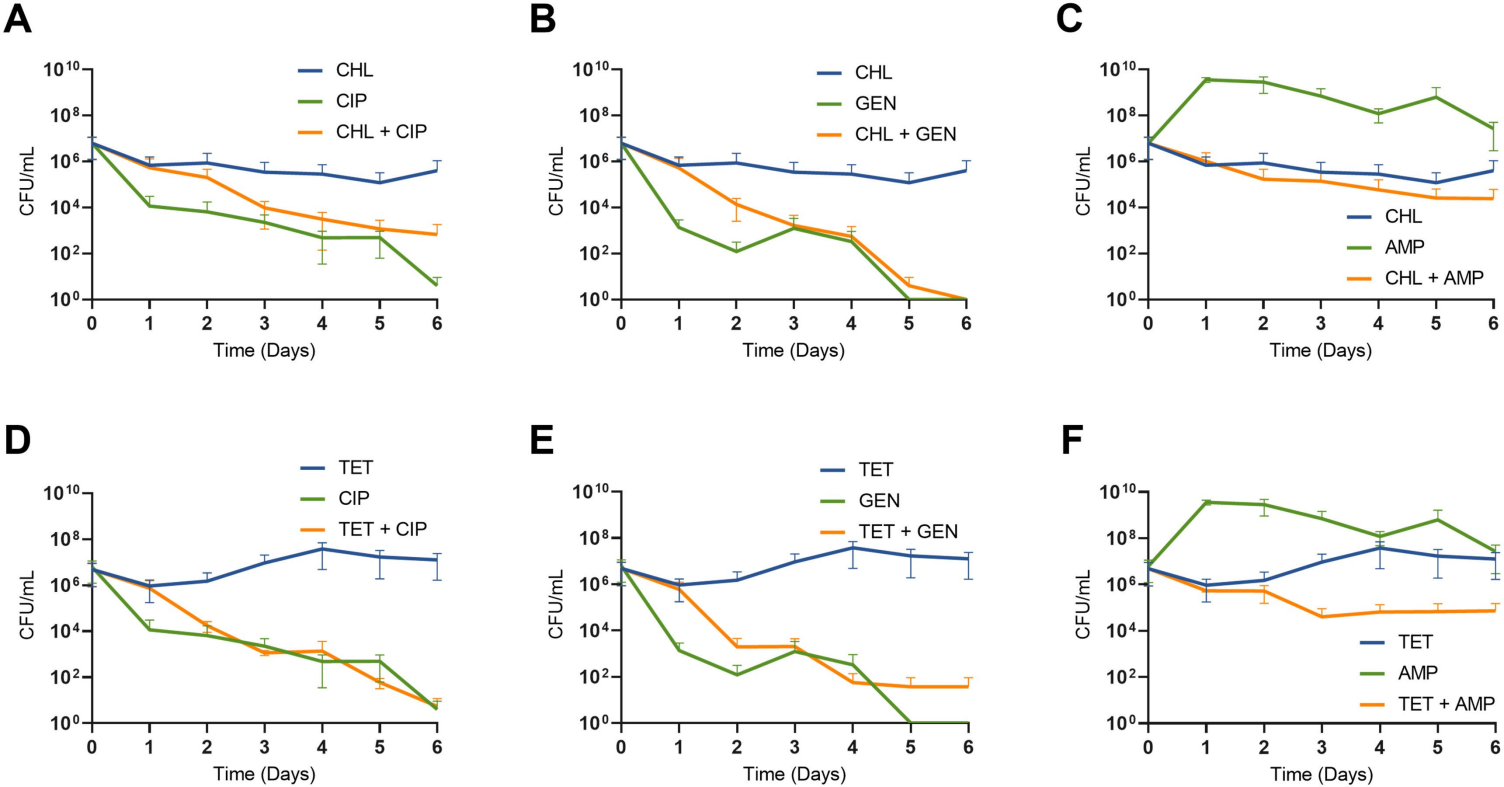
The treatment of *S. aureus* with bacteriostatic followed by bactericidal antibiotics. Shown are bacterial densities in CFU/mL, each point represents the mean and standard deviation of three biological replicates. Cultures were treated with either chloramphenicol or tetracycline at day 0 before the subsequent addition of a bactericidal antibiotic at day 1 (A–F). Cultures were sampled every day for 6 days and are not transferred. Chloramphenicol is at 4× MIC, tetracycline at 10× MIC, and the bactericidal drugs are all at 12× MIC. The value of the area under the curve of cultures treated with chloramphenicol alone versus chloramphenicol and then ampicillin is significantly different by an unpaired two-tail *t*-test (***P < 0.001). The value of the area under the curve of cultures treated with ampicillin alone versus chloramphenicol and then ampicillin is significantly different by an unpaired two-tail *t*-test (****P < 0.0001). The value of the area under the curve of cultures treated with tetracycline or ampicillin alone versus tetracycline and then ampicillin is significantly different by an unpaired two-tail *t*-test (****P < 0.0001).

When a clinical isolate of *S. aureus* was treated first with either chloramphenicol or tetracycline followed by a bactericidal drug, the results obtained are qualitatively like those obtained with *E. coli* (Fig. 3). Interestingly, the exposure to bacteriostatic drugs followed by ampicillin led to significantly greater culture clearance than ampicillin alone. While exposure to bacteriostatic drugs followed by gentamicin or ciprofloxacin resulted in high levels of bacterial killing, but slowed down the dynamics of both bactericidal drugs.

Here, 0- and 6-day supernatants treated with either chloramphenicol, azithromycin, tetracycline, ciprofloxacin, gentamicin, or ampicillin were filtered, and the amount of antibiotic present tested through a disk diffusion assay. The diameter of the inhibition zone at both time points was the same for the five supernatants, confirming the stability of the five antibiotics over the course of the 6-day experiments.

Using the Bliss independence null model to assess whether bacteriostatic and bactericidal antibiotics interact synergistically, additively, or antagonistically (12, 13). We found that all tested combinations in *E. coli* and *S. aureus* exhibited synergy (Table 1).

**TABLE 1:** Antibiotic combination effects under the Bliss independence model

Bacteria	Combination	Expected Survival	Actual Survival
*E. coli*	Chloramphenicol and ciprofloxacin	0.7605	0.0181
*E. coli*	Chloramphenicol and gentamicin	0.7605	0
*E. coli*	Chloramphenicol and ampicillin	0.7605	0.0004
*E. coli*	Azithromycin and ciprofloxacin	0.7605	0.0005
*E. coli*	Azithromycin and gentamicin	0.7605	0
*E. coli*	Azithromycin and ampicillin	0.7605	0
*S. aureus*	Chloramphenicol and ciprofloxacin	0.0640	0.0001
*S. aureus*	Chloramphenicol and gentamicin	0.0640	0
*S. aureus*	Chloramphenicol and ampicillin	4.0948	0.0039
*S. aureus*	Tetracycline and ciprofloxacin	2.5918	0
*S. aureus*	Tetracycline and gentamicin	2.5918	0
*S. aureus*	Tetracycline and ampicillin	4.2630	0.0148

The *S. aureus* strain used in this study, MN8, encodes a staphylococcal beta-lactamase. We demonstrated that pre-exposure to bacteriostatic drugs inhibits beta -lactamase production, explaining the observed synergy between these drugs and ampicillin. To assess this effect, *S. aureus* MN8 was incubated with chloramphenicol for 24 hours, after which the cell-free supernatant was collected and used as a growth medium to evaluate the susceptibility of a chloramphenicol-resistant *E. coli* O157:H7 to ampicillin. The MIC of ampicillin for *E. coli* O157:H7 was 8 µg/mL, consistent with results obtained when using supernatants from *S. aureus* cultures grown with chloramphenicol. In contrast, when cell-free supernatants from *S. aureus* cultures incubated without the bacteriostatic drug were used, the MIC of ampicillin for *E. coli* O157:H7 increased to >64 µg/mL. This finding confirms that, in the absence of bacteriostatic drug pre-exposure, the beta-lactamase secreted by *S. aureus* effectively degrades ampicillin, reducing its efficacy, while the pre-exposure to a bacteriostatic drug inhibits the production of this enzyme.

### Effect of treatment order

**FIG 4:**
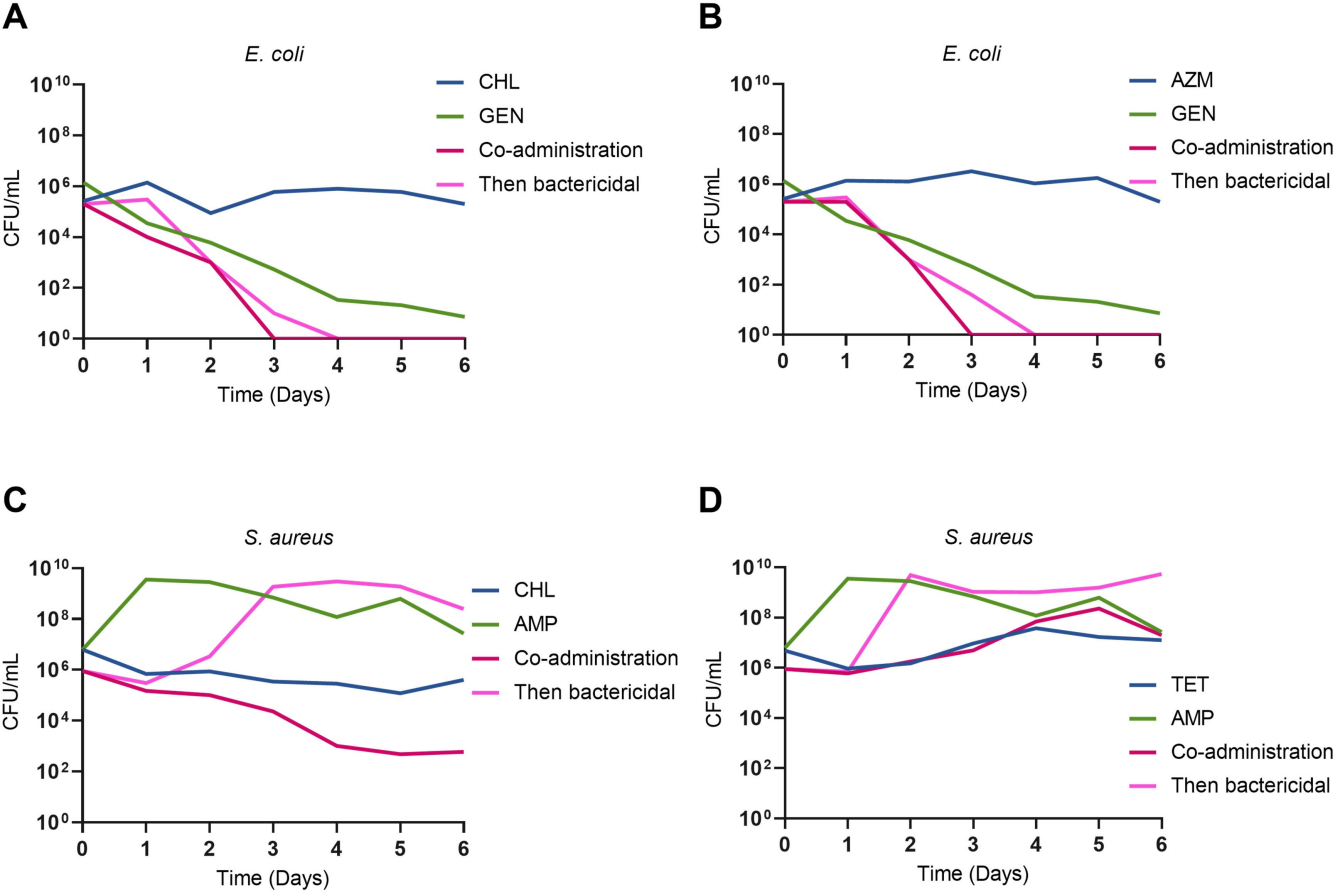
The treatment of *E. coli* and *S. aureus* with co-administration of bacteriostatic and a bactericidal drug, as well as sequential administration of a bactericidal drug after the removal of the bacteriostatic drug. Shown are bacterial densities in CFU/mL (A–D). First, cultures were treated with either chloramphenicol, azithromycin, or tetracycline in combination with gentamicin or ampicillin at day 0 and were sampled every day for 6 days without transfer. Second, cultures were treated with either chloramphenicol, azithromycin, or tetracycline at day 0 before cleaning the cells and the subsequent addition of a bactericidal antibiotic at day 1. Cultures were sampled every day for 6 days and not transferred. Chloramphenicol is at 4× MIC, azithromycin at 4× MIC tetracycline at 10× MIC, gentamicin at 4× MIC, and ampicillin at 12× MIC.

Over the course of the bacteriostatic then bactericidal experiments, we observed that over a 6-day period the sequential administration of gentamicin following bacteriostatic pre-exposure in *E. coli* led to faster bacterial clearance compared to gentamicin alone. To further investigate this effect, we tested the co-administration of chloramphenicol (Fig. 4A) or azithromycin (Fig. 4B) with gentamicin, as well as subsequent exposure wherein cultures are treated with chloramphenicol (Fig. 4A) or azithromycin (Fig. 4B), the bacteriostatic drug is removed and then treated with gentamicin. Our findings confirmed that in all scenarios, the presence of a bacteriostatic drug effectively prevented the emergence of gentamicin-resistant small colony variants.

Similarly, when the clinical isolate of *S. aureus* was treated with either chloramphenicol or tetracycline followed by ampicillin, culture clearance was greater than with ampicillin alone. However, the co-administration of bacteriostatic drugs with ampicillin as well as sequential treatment with removal of the bacteriostatic drug and then treatment with ampicillin produced clearance dynamics similar to those observed with ampicillin alone (Fig. 4C and D). The only exception was the co-administration of chloramphenicol and ampicillin, which resulted in enhanced bacterial clearance (Fig. 4C).

## DISCUSSION

Contrary to the maxim that bacteriostatic drugs are completely antagonistic to bactericidal agents (4), our findings indicate that bactericidal action does occur after bacteriostatic pre-exposure and that sequential use of bacteriostatic and bactericidal antibiotics is feasible and potentially beneficial. In neither *E. coli* nor *S. aureus*, pre-exposure with bacteriostatic antibiotics was antagonistic to the subsequent action of bactericidal drugs. Quite to the contrary, pre-exposure with bacteriostatic drugs was synergistic and in several cases allowed for a higher degree of killing than exposure with just the bactericidal agents alone. Two key observations emerged from our study. First, bacteriostatic pre-exposure effectively suppressed the emergence of gentamicin-resistant small colony variants in *E. coli* that ascend with the exposure of gentamicin alone, increasing the rate of bacterial clearance (14). Second, in the case of *S. aureus* MN8, an inducible beta-lactamase producer, pre-exposure with bacteriostatic drugs facilitated better control of bacterial density by ampicillin, although a significant population of what are likely traditional persister cells remained. We hypothesize this occurs due to the slowing of bacterial growth and metabolism with a bacteriostatic agent, the bacteria does not produce sufficient beta-lactamase, limiting the enzyme’s ability to inactivate ampicillin. In this scenario, while bacteriostatic pre-exposure helped ampicillin more effectively reduce bacterial density, persister cells, which are known to be tolerant to antibiotics due to their dormant state, persisted and may continue to pose a challenge for complete bacterial clearance. These results are consistent with the concept of cellular hysteresis (a persistent change in bacterial physiology, which is induced by one antibiotic and enhances susceptibility toward another antibiotic) and open the door to looking at collateral sensitivity with parings of bacteriostatic and bactericidal antibiotics (15–18). However, our experimental design does not mirror that traditionally used to examine hysteresis, instead our experimental design is more analogous to the clinical realities of antibiotic usage, where one drug is given first and then supplemented with additional antibiotics. Our findings align with previous studies, highlighting that exposure to certain bacteriostatic drugs (such as chloramphenicol, azithromycin, or tetracycline) does not fully inhibit bacterial replication. Instead of completely halting ribosomal activity, these antibiotics appear to permit a form of “selective translation,” allowing the synthesis of certain proteins while impairing overall protein production, which may contribute to the bacteria’s ability to maintain minimal activity despite the presence of these ribosome-targeting drugs (9, 19–21). As demonstrated by our results, bacteriostatic agents that target ribosomal protein synthesis can deplete the strain of resistance mechanisms against bactericidal agents.

Unexpectedly, almost all combinations where a bacteriostatic drug was applied before a bactericidal drug exhibited synergy while combinations with other treatment regimens (e.g., co-administration or bacteriostatic then bactericidal with the removal of the bacteriostatic drug) did not always result in synergy. This result is critically important when considering the vast amount of high-throughput screens for drug-drug synergy and antagonism as these screens almost always look at the co-administration of both agents (12, 22–27).

As with all *in vitro* studies of antibiotics, the role of the host’s immune system was not considered in our experiments, a critical factor in the success of antibiotic therapy (28). One hypothesis regarding why antibiotic therapy is successful *in vivo* is that the drugs slow down growth or reduce the density of bacteria, providing an opportunity for the host’s innate immune response to control and potentially eliminate the infecting bacteria (29). While our study did not achieve complete bacterial clearance in all scenarios, whether through single-drug therapy or sequential bacteriostatic and bactericidal exposures, we suggest that stopping the net growth of bacteria and achieving a higher degree of clearance over time would be sufficient for the host’s immune system to control the residual bacterial population effectively. Subsequently, it is essential to further explore the pharmacodynamics of combined bacteriostatic and bactericidal exposures, both *in vitro* and *in vivo*, to better design antibiotic treatment protocols.

## MATERIALS AND METHODS

### Growth media

LB broth (244620) was obtained from BD. Muller Hinton II (MHII) Broth (90922–500G) obtained from Millipore. LB agar (244510) for plates was obtained from BD.

### Growth conditions

All experiments were conducted at 37°C and shaken continuously.

### Bacterial strains

*E. coli* MG1655 and the chloramphenicol-resistant *E. coli* O156:H7 were obtained from the Levin Lab Bacterial collection. *S. aureus* MN8 was obtained from Tim Read of Emory University.

### Antibiotics

Ciprofloxacin (A4556) was obtained from AppliChem Panreac, chloramphenicol (23660) was obtained from USB, ampicillin (A9518-25G) was obtained from Sigma-Aldrich, azithromycin (3771) was obtained from TOCRIS, gentamicin (BP918-1) was obtained from Fisher BioReagents, and tetracycline (T17999) was obtained from Research Products International.

### Sampling bacterial densities

The densities of bacteria were estimated by serial dilution in 0.85% saline and plating. The total density of bacteria was estimated on Lysogeny Broth (LB) plates with 1.6% agar.

### Minimum inhibitory concentrations

Antibiotic MICs were estimated using a twofold microdilution procedure on MH as described in and following CLSI guidelines (30).

### Antibiotic killing assays

For these experiments, 24 hours cultures of *E. coli* MG1655 or *S. aureus* MN8 were added to LB broth or MHII at an initial density of approximately 10^6^ cells per mL, followed by a 24 hour incubation with a bacteriostatic drug at the concentrations indicated in the figure captions. After the 24 hour incubation, a bactericidal antibiotic was added in the same culture flask at the concentrations indicated in the figure captions. The cultures containing both drugs were incubated for 5 days without transferring. Bacterial densities were estimated before the addition of the bacteriostatic drugs (*t* = 0), before the addition of the bactericidal drug (*t* = 1), and on each subsequent day.

### Antibiotic stability

Initial (day 0) and final (day 6) cultures in the presence of ciprofloxacin, chloramphenicol, ampicillin, azithromycin, gentamicin, or tetracycline were centrifuged and the supernatants filtrated. Here, 20 μL of these supernatants were spotted on a seven mm blank disk, and MG1655 and MN8 were used as the lawn. After 24  hour incubation, the zone of inhibition was recorded.

### Statistics

The growth rate was analyzed using GraphPad Prism, calculating the area under the OD600 nm versus time curve. Measurements were made in three independent replicates for each condition. Statistical significance analysis was carried out by paired *t*-tests using GraphPad Prism (version 10.2.0).

Antibiotic synergy was assessed by comparison with a Bliss null model, for which the expected additive combination effects were obtained by multiplication of the survival fractions of the antibiotics acting alone.

## Data Availability

All data are available in this report.
